# Comparison of the Metabolic Profiles Associated with Protonitazene and Protonitazepyne in Two Severe Poisonings

**DOI:** 10.3390/metabo15060371

**Published:** 2025-06-05

**Authors:** Romain Magny, Thomas Schiestel, Aymen M’Rad, Bertrand Lefrère, Jean-Herlé Raphalen, Stanislas Ledochowski, Laurence Labat, Bruno Mégarbane, Pascal Houzé

**Affiliations:** 1 Laboratoire de Toxicologie, Fédération de Toxicologie, Hôpital Lariboisière, AP-HP, 75010 Paris, France; thomas.schiestel@aphp.fr (T.S.); laurence.labat@aphp.fr (L.L.); pascal.houze@aphp.fr (P.H.); 2 INSERM, Université Paris-Cité, Optimisation Thérapeutique en Neuropharmacologie OTEN U1144, 75006 Paris, France; bruno.megarbane@aphp.fr; 3 Réanimation Médicale et Toxicologique, Fédération de Toxicologie, Hôpital Lariboisière, AP-HP, 75010 Paris, France; aymen.mrad@aphp.fr; 4 Service de Biochimie Générale, Hôpital Necker-Enfants Malades, AP-HP, 75015 Paris, France; bertrand.lefrere@aphp.fr; 5 Department of Anesthesiology and Intensive Care Medicine, Adult Intensive Care Unit, Necker Hospital, 75015 Paris, France; jena-herle.raphalen@aphp.fr; 6 Service de Réanimation Polyvalente, Médipôle Lyon-Villeurbanne, Ramsay Santé, 69100 Villeurbanne, France; sledochowski@scprea.fr

**Keywords:** protonitazene, nitazene, new synthetic opioid, high resolution mass spectrometry, molecular network

## Abstract

Nitazenes represent an emerging class of new synthetic opioids characterized by a high-potency μ-opioid receptor (MOR) agonist activity. **Background**: We report two 20-year-old males who presented with severe neurorespiratory depression with typical opioid syndrome, but no opioid identification based on routine blood and urine screening tests. The first patient recovered with supportive care, mechanical ventilation, and naloxone infusion, whereas the second patient developed post-anoxic cardiac arrest and died from brain death. **Methods**: A complementary comprehensive toxicological screening using liquid chromatography coupled with high-resolution mass spectrometry (LC-HRMS) was performed, and data were processed using a dedicated molecular network strategy to profile the metabolites. **Results**: Protonitazene and protonitazepyne, two nitazenes differing in their ethylamine moieties (i.e., a diethyl versus a pyrrolidine substitution, respectively), were identified. We found an extensive metabolism of protonitazene, leading to the identification of multiple phase I (resulting from hydroxylation, N-desethylation, and O-despropylation) and phase II (resulting from glucuronidation) metabolites. By contrast, protonitazepyne metabolism appeared limited, with one metabolite annotated confidently, protonitazepyne acid, which resulted from the oxidative pyrrolidine ring cleavage. **Concusions**: To conclude, nitazene detection is highly challenging due to its extensive structural and metabolic diversity. Our findings highlight the contribution of the untargeted LC-HRMS screening approach and suggest that diagnostic product ions can serve as robust markers for nitazene identification.

## 1. Introduction

Nitazenes, known as 2-benzylbenzimidazoles, are new synthetic opioids developed in the 1950s as potential therapeutic agents due to morphine-like analgesic properties [1,2]. Despite a unique chemical structure with promising potential, no compound has ever received marketing authorization due to concerning high potency, toxicity, and addictive liability [2]. Nitazenes act as highly potent μ-opioid receptor (MOR) agonists. Etonitazene, first described in 1957, is, for instance, 100–1000 times more potent than morphine [3,4]. The high affinity of nitazenes to MOR contributes to their extreme toxicity, including neurorespiratory depression and fatality risk, particularly in non-tolerant individuals [4,5].

Nitazenes rapidly gained popularity as illicit recreational drugs [2]. The use of isotonitazene was first reported in 2019 in Europe and the United States [2,6]. Following regulatory controls, isotonitazene was substituted in the market with metonitazene in 2020 and recently with protonitazene [5,7,8]. Such a rapid diversification in nitazene analogs was in agreement with the adaptive strategy developed in the new psychoactive substance market, representing major challenges for toxicologists and regulatory authorities.

The structure-activity relationship of nitazenes has been extensively described [9,10,11,12,13,14]. Nitazenes share a common chemical structure consisting of a benzimidazole core linked to a 2-benzyl substituent and a 1-ethylamine group, required to bind to the MOR [9,12]. A key feature of nitazenes is the 5-nitro group on the benzimidazole ring, which acts as an electron-withdrawing group, enhancing electrophilicity and contributing to MOR binding [12,15]. The structural modifications of nitazenes are usually observed at the phenyl moiety, the ethylamine group, and the position of the nitro group. Substitutions on the phenyl ring, such as a propoxy (as in protonitazene), a methoxy, or halogen groups (e.g., chlorine or fluorine), enhance lipophilicity [9]. In the metonitazene family, while the N-diethyl and N-pyrrolidine analogs exhibit similar affinities to the MOR, the N-desethyl analog has a 10-fold lower affinity, supporting the contribution of the tertiary amine in the interaction with the MOR [12]. The nitro group on the benzimidazole ring is additionally crucial, as suggested by alterations in the para-positioned nitro group, present in most nitazenes, that result in reduced potency [15].

While strongly affecting the activity, changes in the structure make the detection and identification of nitazenes in biological matrices difficult. Analytical challenges include (*i*) the growing number of structural analogs emerging on the market, (*ii*) the extensive metabolism in humans, and (*iii*) the extremely low concentrations (in the ng/mL range) commonly found in biological samples, which require highly sensitive analytical methods [3,16,17]. Targeted approaches developed to profile nitazenes in biological samples may quickly become ineffective given the emergence of new structures [3,16,17,18]. Interestingly, in vitro models using human liver microsomes and primary human hepatocytes used to predict the metabolic profiles of nitazenes confirmed extensive phase I metabolism, such as N- and O-dealkylation, hydroxylation, and reduction [19,20,21,22]. However, discrepancies between metabolites identified in vitro and from human samples suggested different pathways, such as conjugation reactions (phase II) occurring in vivo but not in vitro. Therefore, the development of sensitive and specific untargeted analytical methods is essential to enable the detection and identification of multiple nitazene analogs and metabolites in human samples. Liquid chromatography coupled with high-resolution mass spectrometry (LC-HRMS) is the gold standard approach, and non-targeted screening approaches combined with computational strategies such as molecular networking allow visualization, clustering, and identification of structurally related metabolites in the complex matrices [23,24,25,26,27,28].

**Figure 1:**
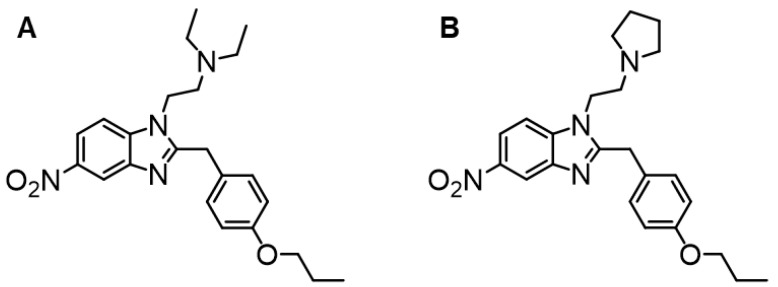
Chemical structure of (**A**) protonitazene and (**B**) protonitazepyne.

Here, we investigated two poisonings related to exposure to protonitazene and protonitazepyne, which involve a diethylamine and pyrrolidine substitution as ethylamine moieties, respectively (Figure 1). We performed a non-targeted LC-HRMS approach, completed with a molecular network analysis of plasma and urine samples collected at the hospital.

## 2. Case Reports

**Patient 1.** A 20-year-old male with a history of depression, regular cannabis use, and autism spectrum disorder treated with clomipramine and alprazolam, was admitted to the intensive care unit with coma (Glasgow coma score, 3), pinpoint myosis, and deep respiratory acidosis. An unknown powder was found alongside but was not retrieved during the patient’s management, and therefore could not be subjected to analysis. He was mechanically ventilated for 24 h, and then extubated on naloxone infusion. He was transferred to the psychiatric ward. Routine targeted toxicological blood analysis identified clomipramine (116 ng/mL, therapeutic range, 100–200 ng/mL) and tetrahydrocannabinol (THC) and its metabolites. These molecules were also identified in the investigated urine sample. Using an untargeted screening approach as fully described hereafter, protonitazene was identified and quantified at 6.2 and 9.0 ng/mL in plasma and urine, respectively.

**Patient 2.** A 20-year-old man with a history of depression treated with cyamemazine, olanzapine, and valproic acid was found unresponsive by his mother. On the emergency medical service’s arrival, the patient was in asystole with an elevated end-tidal carbon dioxide of >110 mmHg. He was promptly resuscitated, mechanically ventilated, and transported to the intensive care unit. On day 3, despite relative cardiovascular stability, brain death was diagnosed. Routine targeted toxicological blood analysis identified cyamemazine (10 ng/mL, therapeutic values < 70 ng/mL) and THC and its metabolites, also found in urine, with cocaine and benzoylecgonine. The untargeted toxicological analysis allowed the identification of protonitazepyne, which was then quantified at 10.7 and 3.1 ng/mL in plasma and urine, respectively.

Noteworthy, a six-month period elapsed between Case 1 and Case 2, which were identified in late 2023 and in 2024, respectively.

## 3. Materials and Methods

### 3.1. Chemicals and Reagents

All reference compounds in powder or at a concentration of 1 mg/mL in methanol were purchased from LGC Group (Molsheim, France), including protonitazene and protonitazepyne. Alprazolam-d5, amphetamine-d5, buprenorphine-d4, MDEA-d5, EDDP-d3, methadone-d3, morphine-d3, and propranolol-d7 at the concentration of 0.1 mg/mL in methanol were obtained from LoGiCal^®^ (LGC GmBH, Molsheim, France). Ultrapure water, acetonitrile (ACN), isopropanol (IPA), methanol (MeOH), formic acid (FA), ammonium formate, and other chemical reagents, all with a purity greater than 99% suitable for HRMS, were obtained from Fischer Chemicals™ (Illkirch, France).

### 3.2. Qualitative and Quantitative Untargeted Toxicological Screening Using LC-HRMS/MS

#### 3.2.1. Sample Preparation

A volume of 150 µL of LC-MS grade water, 350 µL of acetonitrile, containing the internal standards (alprazolam-d5, amphetamine-d5, buprenorphine-d4, MDEA-d5, EDDP-d3, methadone-d3, morphine-d3 and propranolol-d7; at a concentration of 10 ng/mL) and 350 µL of isopropanol were added to 100 µL of urine or plasma samples. Samples were quickly vortexed and then kept at −20 °C for 1 h to precipitate proteins. The precipitated proteins were pelleted by centrifugation at 14,000 rpm for 10 min. Supernatants were collected and solvents evaporated at 40 °C under a gentle stream of nitrogen. Dry residues were dissolved in 100 µL H_2_O/MeOH (50/50%, *v*/*v*). A volume of 10 µL of the extracts was injected into the analytical system.

#### 3.2.2. Analytical Conditions

The LC-ESI-HRMS/MS system was based on a Vanquish^®^ LC pump (ThermoFisher Scientific, Bremen, Germany) and autosampler coupled to an Exploris^®^ 120 mass spectrometer (ThermoFisher Scientific, Bremen, Germany) equipped with a heated electrospray ionization (HESI) probe operating in positive ion mode. The analytical system was managed using TraceFinder^®^ 4.0 software (ThermoFisher Scientific). LC was performed on an Accucore^®^ Phenyl Hexyl (100 × 2.1 mm, 2.6 µm, (ThermoFisher Scientific)) column maintained at 40 °C. The flow rate was set at 500 µL/min. The chromatographic and mass spectrometric conditions have been previously described [23,24,29]. For the routine targeted screening, the acquisition of MS/MS spectra is based on an inclusion list of about 1500 compounds, while for the untargeted one, the acquisition of MS/MS spectra is performed on the three most intense ions at each cycle. In this mode, a full-scan acquisition was performed at a resolution of 120,000, between *m*/*z* 100 to 1000, followed by the acquisition of three MS/MS spectra on the three most intense ions, at a resolution of 17,500 with an isolation window of *m*/*z* 2. The dynamic exclusion mode used was set at 3 s for each selected ion. MS and MS/MS data were managed by Thermo Scientific TraceFinder^®^ 4.1 software.

#### 3.2.3. Data Processing for Untargeted Analysis

Raw data files acquired in polarity switching mode were converted into open-source mzXML files under MSConvert 3.0™. Data processing was performed using MZmine 4.4.3 [30,31] to generate the molecular network, as described [29]. The molecular network was generated using the feature-based molecular networking workflow of the Global Natural Products Social (GNPS) platform and using MetGem software [32,33]. Molecular networks were built using LC-ESI-HRMS/MS data obtained from all analyzed plasma samples, as previously described [29]. The dereplication step of endogenous and exogenous metabolites was obtained using the GNPS database library and an in-house database. The structural assignment of protonitazene, protonitazepyne, and their corresponding metabolites was based on MS and MS/MS data, using a tolerance window of 5 ppm for the exact mass measurement of the precursor ion and 15 ppm for product ions. Annotation was supported by experimental chromatographic retention time (t_R_) values. SIRIUS 6.1.0 was used for molecular formula determination and metabolite identification [34,35].

#### 3.2.4. Quantitative Analysis

To quantify protonitazene and protonitazepyne, a dedicated quantitative method was developed. The same chromatographic and mass spectrometry parameters were used for the quantification of both protonitazene and protonitazepyne. The method was validated according to European Medicine Agency (EMA) guidelines, including repeatability. The intermediate precision was lower than 15% for the three levels of quality control. The method was linear between 0.5 and 20 ng/mL, and the limit of detection was 0.1 ng/mL. Note that no matrix effect was observed in both plasma and urine.

## 4. Results

### 4.1. Analytical Features of Protonitazene and Protonitazepyne

#### 4.1.1. Analytical Features of Protonitazene

**Figure 2:**
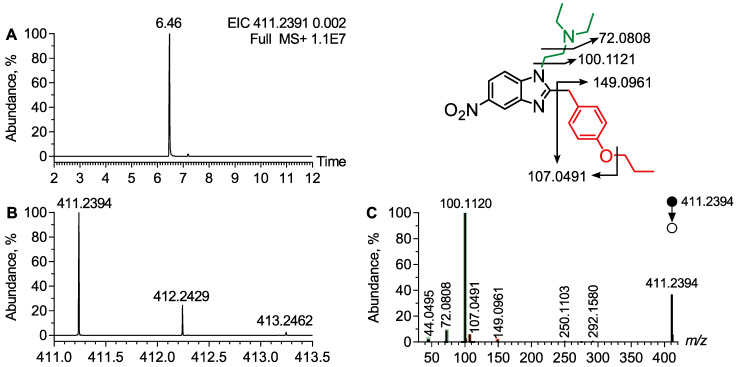
Analytical features of protonitazene. (**A**) Extracted ion chromatogram at *m*/*z* 411.2391 corresponding to the [M + H]^+^ ion of protonitazene from LC-HRMS data obtained following the analysis of plasma extract. (**B**) Experimental isotopic pattern of protonitazene and (**C**) MS/MS spectra of the [M + H]^+^ ion of protonitazene.

Protonitazene was identified at t_R_ = 6.46 min as the [M + H]^+^ ion at *m*/*z* 411.2396, with an isotopic pattern consistent with the molecular formula C_23_H_30_N_4_O_3_ (Figure 2A,B). The MS/MS spectrum at *m*/*z* 411.2396 displayed several diagnostic product ions, providing structural insights into the molecule (Figure 2C). The main product ion, detected at *m*/*z* 100.1120, was attributed to the ethylamine moiety bearing a N,N-diethyl group. This diethylazaridinium ion underwent further fragmentation, losing one or two ethylamine radicals, which were detected at *m*/*z* 72.0808 and *m*/*z* 44.0495, respectively. The N,N-diethyl ethylamine moiety identification of protonitazene was supported by three distinct product ions. A minor product ion at *m*/*z* 292.1570 was assigned to the sequential loss of the diethylamine group and nitro function (Figure 2C). This ion was further fragmented with the loss of the propyl moiety, producing an ion at *m*/*z* 250.1103. Another diagnostic ion at *m/z* 149.0961 was attributed to the propoxyphenyl moiety of protonitazene. This propoxyphenylmethylium ion underwent additional fragmentation by losing the propyl group, leading to a product ion at *m*/*z* 107.0491, confirming the propoxy substitution on the phenyl ring of the molecule. The identification of the phenyl substitution and benzimidazole moieties in protonitazene was supported by diagnostic product ions. The MS/MS spectra revealed fragments corresponding to the ethyldiethylamine, benzimidazole, and propoxyphenyl moieties, providing strong evidence for its identification. These key fragments suggested that molecular networking could cluster protonitazene with its metabolites.

#### 4.1.2. Analytical Features of Protonitazepyne

**Figure 3:**
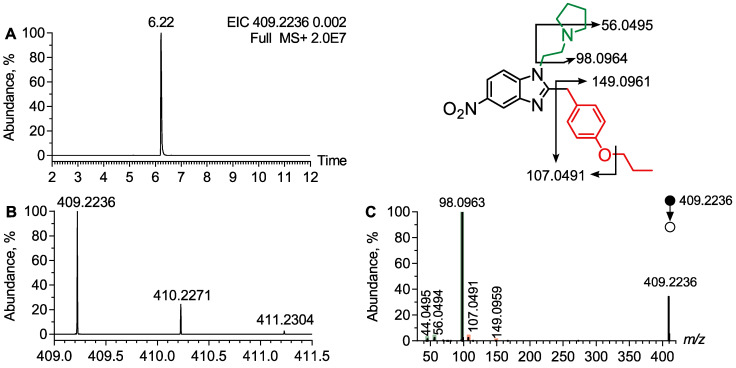
Analytical features of protonitazepyne. (**A**) Extracted ion chromatogram at *m*/*z* 409.2236 corresponding to the [M + H]^+^ ion of protonitazepyne from LC-HRMS data obtained following the analysis of plasma extract. (**B**) Experimental isotopic pattern of protonitazepyne and (**C**) MS/MS spectra of the [M + H]^+^ ion of protonitazepyne.

Protonitazepyne was detected at t_R_ = 6.22 min as the [M + H]^+^ ion at *m*/*z* 409.2234, with an isotopic pattern consistent with the molecular formula C_23_H_28_N_4_O_3_ (Figure 3A,B). The MS/MS spectrum at *m*/*z* 409.2234 displayed a main product ion at *m*/*z* 98.0964 attributed to the ethylamine moiety bearing a N-pyrrolidine group (Figure 3C). A minor product ion at *m*/*z* 250.1103 was attributed to the sequential loss of the pyrrolidine ethylamine group, the nitro function, and the propyl moiety, reflecting the benzimidazole-phenyl core of protonitazepyne. The MS/MS spectra of protonitazepyne exhibited diagnostic ions at *m*/*z* 149.0961 and *m*/*z* 107.0491, useful to characterize the propoxyphenyl moiety of the molecule. These diagnostic product ions highlighted the structural similarity between protonitazene and protonitazepyne while confirming the distinct substitution of the ethylamine moiety. The MS/MS spectra of protonitazepyne provided multiple diagnostic ions to allow structural identification.

### 4.2. Qualitative Metabolite Profiling

We used a dedicated molecular network strategy to profile the metabolites of protonitazene and protonitazepyne. Relying on the product ions displayed on their respective MS/MS spectra, we anticipated our approach to effectively cluster protonitazene and protonitazepyne, together with their respective metabolites.

#### 4.2.1. Metabolite Profiling of Protonitazene

**Figure 4:**
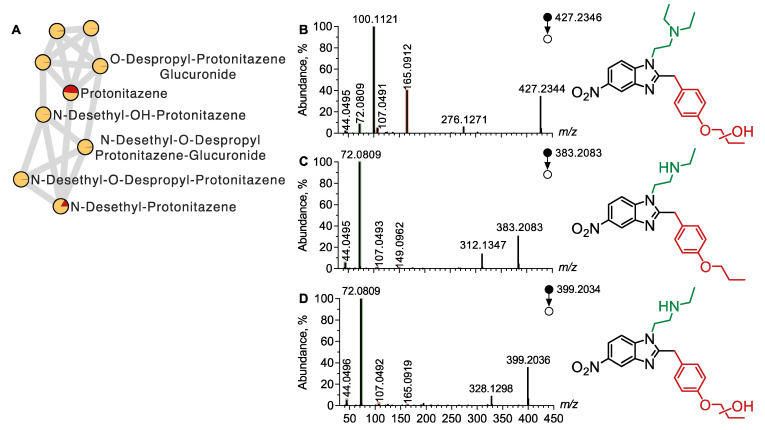
Molecular network of protonitazene metabolites. Molecular network was built using the LC-HRMS data from both plasma and urine extracts. (**A**) Overview of the cluster of protonitazene metabolites. MS/MS spectra of the [M + H]^+^ ion of (**B**) OH-Protonitazene, (**C**) N-Desethyl Protonitazene, and (**D**) N-Desethyl OH-Protonitazene.

A molecular network was built using LC-HRMS/MS data from plasma and urine extracts analyzed in positive ion mode (Figure 4A). In the molecular network, the node corresponding to protonitazene was linked to another node with a mass shift of +15.9951, indicative of a hydroxylation reaction. The MS/MS spectrum of this ion (*m*/*z* 427.2347) revealed diagnostic product ions, including *m*/*z* 100.1121, *m*/*z* 72.0809, and *m*/*z* 44.0495, typical of the N,N-diethyl ethylamine moiety (Figure 4B). Additional product ions, such as *m*/*z* 165.0912, suggested that hydroxylation occurred on the propoxyphenyl moiety of OH-protonitazene. The detection of *m*/*z* 107.0491 additionally supported the hypothesis of a hydroxyphenyl group. Our observations strongly suggested that OH-protonitazene was hydroxylated on the propoxy chain.

The protonitazene node was also connected to another node with a mass shift of −28.0318, corresponding to the loss of an ethyl group. The MS/MS spectrum of this ion (*m*/*z* 383.2083) displayed intense product ions at *m*/*z* 72.0809 and *m*/*z* 44.0495, consistent with the loss of the ethyl group from the ethylamine moiety (Figure 4C). The presence of diagnostic ions for the propoxyphenyl moiety (*m*/*z* 149.0962 and *m*/*z* 107.0493) additionally supported the identification of N-desethyl protonitazene.

Two additional nodes were identified, representing metabolites with combined structural changes. OH-protonitazene and N-desethyl protonitazene were linked to a node with a mass shift of +15.9951 and −28.0318, respectively, consistent with the annotation of N-desethyl OH-protonitazene. The MS/MS spectrum of this compound (*m*/*z* 399.2023) displayed fragments at *m*/*z* 72.0809 and *m*/*z* 44.0495, allowing the identification of the ethylamine moiety, and *m*/*z* 107.0491 and *m*/*z* 165.0909, indicating hydroxylation on the propoxyphenyl group (Figure 4D). An additional node in the cluster was observed at *m*/*z* 341.1613, with a molecular formula consistent with C_18_H_20_N_4_O_3_, suggesting the annotation of N-desethyl O-despropyl protonitazene. The MS/MS spectrum showed fragments at *m*/*z* 72.0809, *m*/*z* 44.0495, and *m*/*z* 107.0491, reinforcing this identification.

Two glucuronide conjugates were detected with precursor ions at *m*/*z* 517.1936 and *m*/*z* 545.2249. The two molecules displayed a neutral loss of 176.0320 Da, allowing the identification of glucuronoconjugation. The MS/MS spectrum at *m*/*z* 517.1936 included product ions at *m*/*z* 341.1610, *m*/*z* 72.0808, and *m*/*z* 107.0493, consistent with the annotation of N-desethyl O-despropyl protonitazene glucuronide. Similarly, the spectrum at *m*/*z* 545.2250 revealed product ions at *m*/*z* 369.1925, *m*/*z* 107.0492, and *m*/*z* 100.1121, supporting its identification as O-despropyl protonitazene glucuronide.

In summary, this untargeted screening approach combined with molecular networking identified four phase I metabolites, including three metabolic processes (e.g., hydroxylation, desethylation, and despropylation) and two phase II glucuronide conjugates of protonitazene in the biological samples of case 1.

#### 4.2.2. Metabolite Profiling of Protonitazepyne

**Figure 5:**
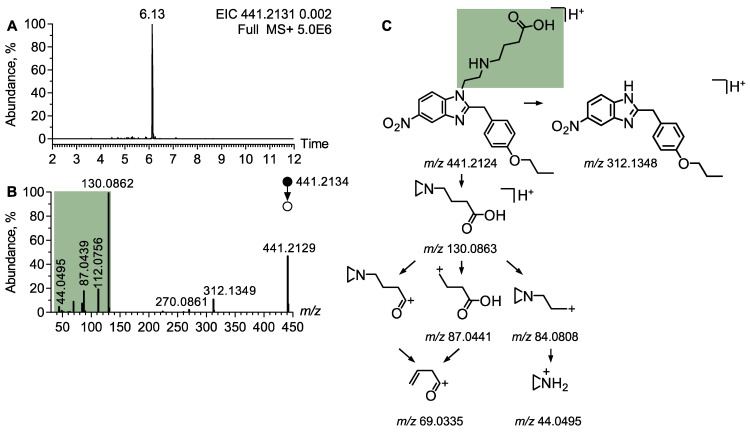
Annotation of protonitazepyne metabolite. (**A**) Extracted ion chromatogram at *m*/*z* 441.2131 corresponding to the [M + H]^+^ ion of protonitazepyne acid from LC-HRMS data obtained following the analysis of a urine extract. (**B**) MS/MS spectra of the [M + H]^+^ ion of protonitazepyne acid. (**C**) Proposed fragmentation pattern of protonitazepyne.

Using the same data processing method, the node corresponding to protonitazepyne was connected to only one other node, with a precursor ion detected at *m*/*z* 441.2129. The isotopic pattern of this ion was consistent with the molecular formula C_23_H_28_N_4_O_5_. The MS/MS spectrum of the ion at *m*/*z* 441.2129 displayed a main product ion at *m*/*z* 130.0862, which was attributed to a butanoic ethylamine chain (Figure 5A). Additional product ions supported the identification of this side chain, including *m*/*z* 112.0752, *m*/*z* 87.0439, and *m*/*z* 69.0335, corresponding to the sequential losses of H_2_O and/or ethylamine. The ion at *m*/*z* 84.0806 provided additional evidence for a terminal carboxylic acid group on the butanoic ethylamine chain as it corresponded to a loss of CO_2_. This ion was identified as an azaridinylpropanylium ion, supporting the proposed structural modification at the side chain. A detailed fragmentation pathway is proposed in Figure 5C. Based on the inspection of MS/MS spectra, the metabolite was annotated as protonitazepyne acid.

To conclude, regarding the qualitative metabolite profiling of protonitazene and protonitazepyne, Table 1 provides an overview of the experimental [M + H]^+^ *m*/*z* values, retention times, and diagnostic product ions used for the identification of the related metabolites. Noteworthy, these diagnostic ions played a key role in clustering parent compounds and their metabolites within the molecular network. Furthermore, Figure S1 displays the chromatogram of each metabolite of protonitazene metabolite.

### 4.3. Relative Metabolite Profiling

Following the identification of the metabolites of protonitazene and protonitazepyne, we aimed to describe their relative distribution in the biological samples of the two cases, using the area under the curve (AUC) of the chromatographic peak of each annotated metabolite along with its parent compound.

#### 4.3.1. Relative Metabolite Profiling of Protonitazene

**Figure 6:**
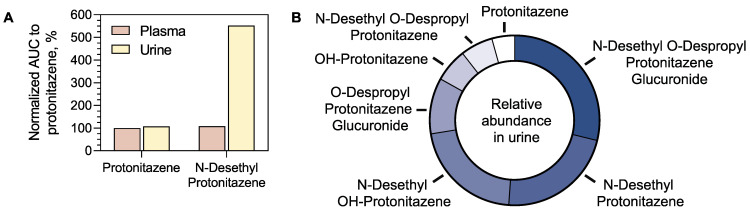
Relative abundance of protonitazene metabolites in urine and plasma. (**A**) Area under the curve of protonitazene and its demethylated metabolites in plasma and urine. (**B**) Relative abundance in the urine of protonitazene metabolites based on the area under the curve of the chromatographic peak.

Protonitazene and its metabolite, N-desethyl protonitazene, were detected in plasma and urine, while N-desethyl protonitazene exhibited a higher intensity in urine (Figure 6A). Based on the AUCs, the most abundant metabolite in urine was N-desethyl O-despropyl protonitazene glucuronide, followed by N-desethyl protonitazene and N-desethyl OH-protonitazene (Figure 6B). Protonitazene AUC was the lowest, suggesting an extensive metabolism of the parent molecule. The relative distribution of protonitazene metabolites in urine is summarized in Figure 6B, showing that phase I and phase II metabolites were predominant over the parent compound.

#### 4.3.2. Relative Metabolite Profiling of Protonitazepyne

While protonitazepyne was readily detected in the plasma and urine of case 2, the annotated metabolite, protonitazepyne acid, was only detected in urine, accounting for almost the whole AUC of the chromatographic peak corresponding to protonitazepyne.

## 5. Discussion

Nitazenes, synthetic opioids structurally distinct from opiates and fentanyl, have emerged as highly potent MOR agonists [4,5]. Nitazenes have gained attraction in the illicit drug market, with a broad array of molecules such as isotonitazene, metonitazene, and protonitazene [2,7]. Their potency, about 10 times the potency of fentanyl, poses the risk of severe respiratory depression and death in overdose, especially in non-tolerant patients. Here, we reported two nitazene poisoning cases with the identification of the parent molecules and their metabolites using an untargeted screening method in plasma and urine.

Manifestations in our two patients were consistent with the potent MOR agonistic activity of nitazenes, responsible for central neurorespiratory depression [36]. Opioid activity of nitazenes was reported to be additionally mediated by β-arrestin2 receptor signaling pathways, which contributes to their enhanced toxicity in comparison to morphine [37]. The two involved nitazenes differ in the substitution of the ethylamine moiety, with the presence of a N,N-diethyl and N-pyrrolidine group in protonitazene and protonitazepyne, respectively. In vitro studies showed comparable potencies at the MOR, with EC_5__0_ values in the low nanomolar range [12], suggesting that the outcome in our patients was more related to differences in the dose and delay of medical intervention rather than in the pharmacological potency of the drugs. Of note, a history of opioid use, including nitazenes, was reported retrospectively in patient 1 but not in patient 2, suggesting a possible additional contribution of the lack of opioid tolerance to patient 2’s death.

Protonitazene and protonitazepyne were found in the ng/mL range in the plasma, consistent with previous reports [3,6,16]. *Postmortem* analyses found peripheral blood protonitazene concentrations ranging from 3.1 to 25 ng/mL [3] and even cases with concentrations as low as 0.1 ng/mL [36,38]. By contrast, to the best of our knowledge, no blood or urine protonitazepyne quantification in poisoning has been published. One study described the use of an analogous nitazepyne of protonitazepyne, namely etonitazepyne, without reported concentrations [39]. Therefore, our study is the first to report a plasma protonitazepyne concentration in a poisoned patient.

While previous studies are based on a targeted quantitative approach focusing on one or several nitazenes, our study relies on a highly sensitive untargeted LC-HRMS approach, allowing for the detection and characterization of both parent compounds and metabolites using limited plasma or urine volumes. The detection of nitazenes in human samples remains challenging, due to their structural diversity. MS/MS spectra of a nitazene molecule are based on a major product ion corresponding to the ethylamine moiety, e.g., N-ethylamine (*m*/*z* 72.0807), N,N-diethylamine (*m*/*z* 100.1120), or N-pyrrolidine (*m*/*z* 98.0960), depending on the substitution. The hydroxyphenyl moiety, a structural part common to all nitazenes, including protonitazene and protonitazepyne, produces a diagnostic ion at *m*/*z* 107.0491. Therefore, screening MS/MS spectra displaying product ions diagnostic of the ethylamine and hydroxyphenyl moieties offers a robust and efficient analytical strategy for identifying nitazenes. Focusing on these key product ions in a non-targeted screening approach with molecular networking data processing is a relevant approach to identifying clusters of interest, a step prior to the structural elucidation of nitazenes and their metabolites.

We identified four phase I and two phase II metabolites of protonitazene. Phase I metabolism included hydroxylation, N-desethylation, and O-despropylation, while phase II involved glucuronidation, consistent with previous data [19,21,38], although the presence of the isopropyl isomer of protonitazene, a metabolite resulting from reduction in the nitro group to an amine on the benzimidazole part of protonitazene or isotonitazene, was not detected in our patient. In a case series including 18 blood and urine samples, this nitro-reduced metabolite was detected inconsistently and at limited amounts in comparison to the parent compound [6], suggesting a case-dependent detection or a limitation by the analytical sensitivity. It must be emphasized that N-desethyl O-despropyl protonitazene glucuronide exhibited the highest AUC in urine, followed by N-desethyl protonitazene and N-desethyl OH-protonitazene. N-desethyl metabolites may thus be regarded as major metabolites, helpful in improving detection sensitivity. We observed comparable AUCs of protonitazene and N-desethyl protonitazene in the plasma, supporting that this metabolite, with a similar potency to fentanyl at the MOR [10], may have contributed to the observed toxic features.

Whereas protonitazene was extensively metabolized, only one metabolite of protonitazepyne was confidently annotated in the urine of patient 2. The manual inspection of high-resolution MS/MS data enabled the identification of protonitazepyne acid, a metabolite resulting from the oxidative cleavage of the pyrrolidine ring to yield the corresponding butanoic acid. This metabolic pathway involves two sequential oxidation steps, ultimately leading to the formation of the acid moiety. To the best of our knowledge, only one study investigated the metabolism of N-pyrrolidine nitazenes, proposing metabolites resulting from hydroxylation, O-desmethylation, and pyrrolidine ring alcohol oxidation from a structurally related compound, etonitazepyne. No acid metabolite was detected, highlighting the discrepancies between in vitro studies using human liver microsomes and human sample-based studies. Interestingly, the opening of the pyrrolidine ring was already reported with other recreative substances such as alpha-pyrrolidinovalerophenone (α-PVP) [40,41]. While awaiting confirmatory studies, our findings highly support that such enzymatic mechanisms, probably based on CYP450 isoforms, may be involved in the metabolism of pyrrolidine-based substances like nitazenes in vivo.

The variability in metabolic profiles of nitazenes emphasizes the challenges of identifying reliable biomarkers of exposure. 4-hydroxy nitazene has been recently proposed to serve as a potential biomarker for nitazenes [6,38]. While detected as a glucuronoconjugated compound in the urine of patient 1, 4-hydroxy nitazene can only be produced from nitazenes bearing an N,N-diethyl group within their ethylamine side chain, making it unsuitable as a universal biomarker for all nitazenes, such as protonitazepyne, which features a pyrrolidine substitution. Therefore, highly sensitive untargeted analytical methods are needed to efficiently detect nitazenes and their metabolites in biological samples. While alternative strategies based on diagnostic ions from the ethylamine and hydroxyphenyl moieties provide an interesting starting point, integrating machine learning-based algorithms with HRMS data could facilitate the automated classification of nitazenes and their metabolites. Future research should focus on developing predictive models capable of distinguishing novel analogs based on fragmentation patterns and metabolic pathways. Implementation of such methods is essential to mitigate false-negative screening results and ensure comprehensive monitoring in the clinical and forensic settings.

It must be noted that this study displays several limitations. As it is based on only two real clinical cases, it limits the generalizability of the findings regarding the metabolic pathways and precludes any robust statistical analysis. Furthermore, the identification and characterization of metabolites relied solely on an untargeted LC-HRMS approach combined with molecular networking, without confirmation by reference standards for all annotated metabolites, and additional metabolites may have remained undetected if not clustered within the molecular network. As biological samples were collected at a single time point during clinical management, this prevented a comprehensive kinetic analysis of the parent compounds and the determination of maximal plasma concentrations, especially since the ingested amount was unknown. Finally, although data processing was carefully performed, minor metabolites present at very low concentrations may have remained undetected.

## 6. Conclusions

Diagnosis of nitazene poisoning is challenging due to ineffective standard targeted screening approaches. Our study confirms the toxicity and provides new insights into the metabolic profiles of protonitazene and protonitazepyne, two structurally related nitazenes. The marked differences we found in the metabolic profiling of these two nitazenes support the need for accurate strategies based on highly sensitive untargeted screening methods such as LC-HRMS combined with molecular networking to identify and monitor the metabolism and distribution of these new synthetic opioids in vivo.

## Figures and Tables

**Table 1:** Nitazene metabolites identification through MS/MS data. The table lists the experimental [M + H]^+^ values, retention times, and key diagnostic ions for each annotated metabolite of protonitazene and protonitazepyne identified in the two investigated cases. Note that the product ion at *m*/*z* 107.0497 is diagnostic of the hydroxyphenyl moiety common to nitazene compounds.

Name	[M + H]^+^	t_R_	Substitution	Diagnostic Product Ions (*m*/*z*)
Protonitazene	411.2391	6.46	N,N-Diethylamine	100.1120, 72.0808, 44.0495
			O-Propyl	149.0961, 107.0491
OH-Protonitazene	427.2347	5.26	N,N-Diethylamine	100.1121, 72.0809, 44.0495
			O-Hydroxy propyl	165.0912, 107.0491
N-Desethyl Protonitazene	383.2083	6.34	N-Ethylamine	72.0808, 44.0495
			O-Propyl	149.0956, 107.0491
N-Desethyl OH- Protonitazene	399.2023	5.08	N-Ethylamine	72.0809, 44.0496
			O-Hydroxy propyl	165.0919, 107.0492
N-Desethyl O-Despropyl Protonitazene	341.1613	4.54	N-Ethylamine	72.0809, 44.0495
			OH	107.0492
O-Despropyl Protonitazene Glucuronide	545.2249	4.05	N,N-Diethylamine	100.1121, 72.0809, 44.0496
			O-Glucuronide	107.0492
N-Desethyl O-Despropyl Protonitazene Glucuronide	517.1936	3.84	N-Ethylamine	72.0808, 44.0495
			O-Glucuronide	107.0492
Protonitazepyne	409.2236	6.22	N-Pyrrolidine	98.0963, 56.0494, 44.0495
			O-Propyl	149.0959, 107.0492
Protonitazepyne acid	441.2131	6.22	N-butanoic acid	130.0862, 112,0757, 87.0441
			O-Propyl	149.0958, 107.0492

## Data Availability

The original contributions presented in this study are included in the article and Supplementary Material. Further inquiries can be directed to the corresponding author.

## Supplementary Materials

The following supporting information can be downloaded at https://www.mdpi.com/article/10.3390/metabo15060371/s1, Figure S1: Extracted ion chromatogram corresponding to the [M + H]^+^ ion of each annotated protonitazene metabolite.
