# Supplementary material for: Comparison of the Metabolic Profiles Associated with Protonitazene and Protonitazepyne in Two Severe Poisonings
Source: Metabolites. 2025 Jun 5;15(6):371. doi: 10.3390/metabo15060371 (PMC12195359; doi:10.3390/metabo15060371)
Supplement: Supplementary file 1 [file metabolites-15-00371-s001.zip › metabolites-3608113-supplementary.pdf]

metabolites

MDPI

Supplementary Materials

Comparison of the Metabolic Profiles Associated with Protonitazene and Protonitazepyne in Two Severe Poisonings

Romain Magny [1,2,*], Thomas Schiestel [1], Aymen M'Rad [3], Bertrand Lefrère [4], Jean-Herlé Raphalen [5], Stanislas Ledochowski [6], Laurence Labat [1,2], Bruno Mégarbane [2,3] and Pascal Houzé [1,2]

[1] Laboratoire de Toxicologie, Fédération de Toxicologie, Hôpital Lariboisière, AP-HP, 75010 Paris, France; thomas.schiestel@aphp.fr (T.S.); laurence.labat@aphp.fr (L.L.); pascal.houze@aphp.fr (P.H.)
[2] INSERM, Université Paris-Cité, Optimisation Thérapeutique en Neuropharmacologie OTEN U1144, 75006 Paris, France; bruno.megarbane@aphp.fr
[3] Réanimation Médicale et Toxicologique, Fédération de Toxicologie, Hôpital Lariboisière, AP-HP, 75010 Paris, France; aymen.mrad@aphp.fr
[4] Service de Biochimie Générale, Hôpital Necker-Enfants Malades, AP-HP, 75015 Paris, France; bertrand.lefrere@aphp.fr
[5] Department of Anesthesiology and Intensive Care Medicine, Adult Intensive Care Unit, Necker Hospital, 75015 Paris, France; jena-herle.raphalen@aphp.fr
[6] Service de Réanimation Polyvalente, Médipôle Lyon-Villeurbanne, Ramsay Santé, 69100 Villeurbanne, France; sledochowski@scprea.fr
* Correspondence: romain.magny@aphp.fr

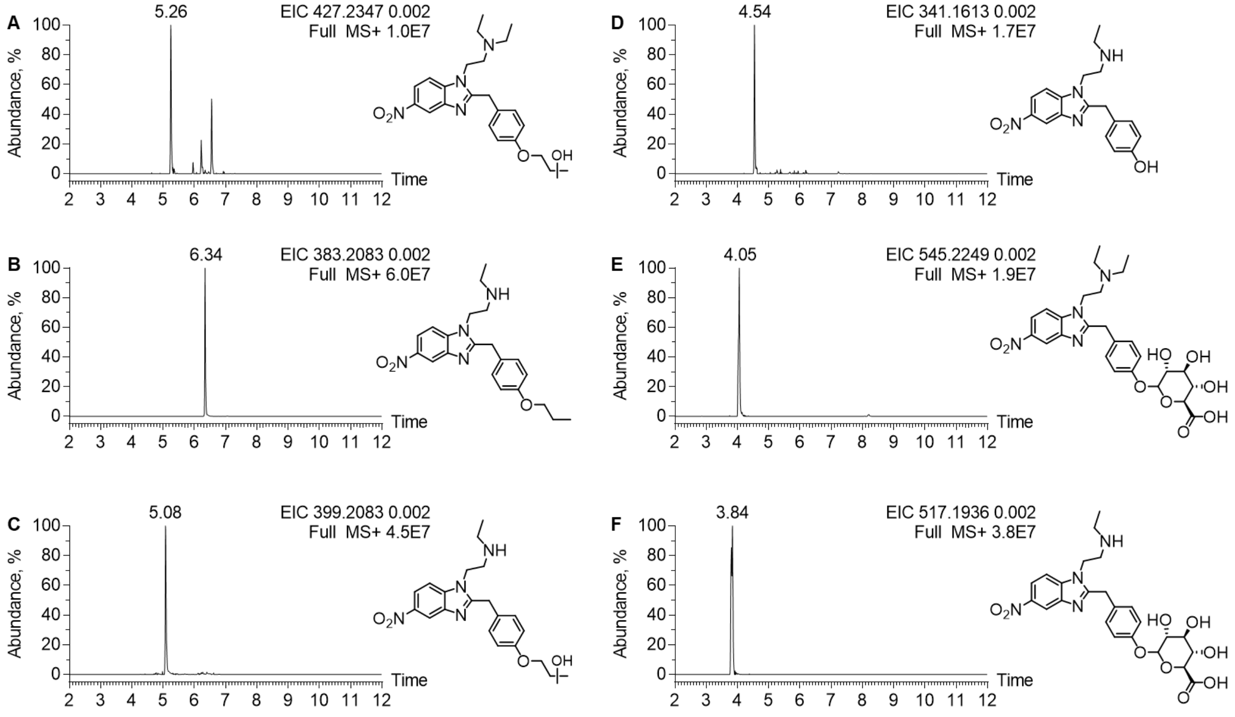

Figure S1. Extracted ion chromatogram corresponding to the [M+H]$^{+}$ ion of each annotated protonitazene metabolite. (A) Hydroxy-Protonitazene, (B) N-Desethyl Protonitazene, (C) N-Desethyl OH-Protonitazene, (D) N-Desethyl O-Despropyl Protonitazene, (E) O-Despropyl Protonitazene Glucuronide, (F) N-Desethyl O-Despropyl Protonitazene Glucuronide.
